# Atopic dermatitis and the risk of osteoporosis and fractures: a meta-analysis of cohort studies

**DOI:** 10.1080/07853890.2025.2607193

**Published:** 2026-01-08

**Authors:** Hongfei Liu, Huilin Ru, Peng Yu, Wei Wei, Shixuan Wang

**Affiliations:** ^a^ The Second Clinical College, Liaoning University of Chinese Medicine, Shenyang, China; ^b^ Department of Dermatology, The Affiliated Hospital of Liaoning University of Chinese Medicine, Shenyang, China; ^c^ Department of Orthopaedics, The First Affiliated Hospital of Henan University of Chinese Medicine, Zhengzhou, China; ^d^ Department of Orthopaedics, The Second Affiliated Hospital of Liaoning University of Chinese Medicine, Shenyang, China

**Keywords:** Atopic dermatitis, cohort study, fracture, meta-analysis, osteoporosis

## Abstract

**Background:**

This meta-analysis aims to evaluate the risk of osteoporosis and fractures in patients with atopic dermatitis (AD) by synthesizing data from cohort studies. We also provide a comprehensive analysis of fracture risks across different severities of AD and anatomical sites.

**Methods:**

Following the PRISMA 2020 guidelines, a systematic search was conducted in PubMed, Embase, and the Cochrane Library up to May 30, 2025. Studies that investigated the relationship between AD and osteoporosis or fractures were included in the analysis. Data extraction and screening were performed independently by two reviewers. Study quality was assessed using the Newcastle-Ottawa Scale (NOS). A random-effects meta-analysis was applied, alongside sensitivity and subgroup analyses. Publication bias was evaluated using funnel plots and Egger’s test.

**Results:**

Ten cohort studies, involving 368 to over 2 million AD patients, were included. NOS scores ranged from 7 to 8, indicating generally high study quality. The pooled analysis revealed a 56% increased risk of osteoporosis (OR = 1.56, 95% CI: 1.14–2.13; *I*^2^ = 99.9%, *p* < 0.0001) and an 8% increased risk of all-cause fractures (OR = 1.08, 95% CI: 1.05–1.10; *I*^2^ = 82.1%, *p* < 0.0001) in AD patients. Subgroup analyses demonstrated a progressive increase in fracture risk with the severity of AD. Specific risks were significantly higher for vertebral fractures (OR = 1.14, 95% CI: 1.08–1.20; *I*^2^ = 67.3%, *p* = 0.009) and lower limb fractures (OR = 1.11, 95% CI: 1.08–1.13; *I*^2^ = 65.0%, *p* = 0.014). Sensitivity analyses confirmed the robustness of these findings, and no significant publication bias was detected (*p* = 0.316).

**Conclusion:**

AD is associated with an increased risk of osteoporosis and fractures, particularly among patients with severe AD and those experiencing vertebral or lower limb fractures. These findings highlight the importance of targeted bone health monitoring in the clinical management of AD patients.

**Registration**: (PROSPERO: CRD420251066550)

## Key Messages

- Atopic dermatitis (AD) is associated with a 56% increased risk of osteoporosis and an 8% increased risk of all-cause fractures.
- The risk of fracture increases progressively with AD severity, and is particularly elevated for vertebral and lower limb fractures.
- Clinicians should be aware of the skeletal complications of AD and consider targeted bone health monitoring, especially in patients with moderate to severe AD.

## Introduction

Atopic dermatitis (AD) is the most prevalent chronic inflammatory skin disorder worldwide, characterized by severe itching, recurrent eczematous lesions, and a variable disease course [1,2]. In 2021, the global prevalence of AD reached 129 million cases, and the number is projected to increase steadily over the next three decades, presenting a significant public health challenge [3]. The pathogenesis of AD is multifactorial, involving genetic predisposition, dysregulated innate immunity, impaired epithelial barrier function, skin microbiome alterations, and environmental factors [4]. In addition to its frequent comorbidity with other atopic conditions, such as allergic rhinitis and asthma [5], AD has also been identified as an independent risk factor for a variety of non-atopic diseases, including cardiovascular and psychiatric disorders [6].

Recent evidence suggests that AD may have a detrimental impact on bone health, primarily through mechanisms such as chronic inflammation [7,8], prolonged corticosteroid use [9], and lifestyle factors like reduced physical activity due to constant itching [10]. A previous meta-analysis by Wu et al. [11] examined the relationship between AD, bone mineral density, osteoporosis, and fracture risk, focusing on both children and adults. However, this analysis included only four studies and was limited in its ability to address publication bias and the differential fracture risks at various anatomical sites. Another meta-analysis by Zhou et al. faced similar limitations, underscoring the need for a more comprehensive examination of this issue [12]. Given the growing body of research, including several large-scale cohort studies conducted recently [13–16], the association between AD and bone health remains controversial. In light of this, we conducted a meta-analysis based on cohort studies to better clarify the risks of osteoporosis and fractures in individuals with AD, ultimately aiming to inform targeted clinical prevention strategies.

## Methods

This meta-analysis was conducted in accordance with the PRISMA 2020 guidelines for systematic reviews [17]. The protocol for this review was prospectively registered with PROSPERO (CRD420251066550), adhering to the established guidelines for systematic reviews and meta-analysis [18].

### Data sources

A comprehensive literature search was performed in three major databases (PubMed, EMBASE, and the Cochrane Library), covering all available studies from database inception to May 30, 2025. No restrictions were imposed based on publication language or date. We employed subject terms (Emtree in Embase, MeSH in PubMed) along with relevant keywords to capture studies related to AD, osteoporosis, fractures, and their variations. To minimize the risk of missing eligible studies, we also examined the reference lists of included studies and relevant prior meta-analyses [11,12]. Detailed search strategies for each database are provided in Supplementary Tables 1–3.

**Table 1.** Basic characteristics of included studies.

Author	Year	Study type	Country	Data source	Enroll period	Follow-up years (mean ± SD) y	AD1 diagnostic criteria	AD severity classification	Number of AD patients	Fracture/Osteoporosis diagnostic criteria	Number of fracture cases	Number of control group	Age (mean ± SD)	Confounders adjusted
Chiesa	2025	Retrospective cohort study	UK	The Health Improvement Network	1994–2015	5.51–5.64 (4.22–4.40)	NR	Treatment-based^13^	465,622	NR	NR	2,678,888	18–65: 37–40 (27–52) y	Age, BMI, smoking status, drinking status and Townsend Index.
						6.21–6.93 (4.88–5.37)			159479				≥ 65: 73–75 (69–81) y	
Hsiao	2024	Retrospective cohort study	China	Taichung Veterans General Hospital, Taichung, Taiwan	2020–2021	NR	Medical records	NR	50	BMD^2^ examination and medical records	13	386	Elderly people: 70 (62, 77) y	Age, corticosteroids use, history of fracture, chronic kidney disease, cardio vascular disease, and chronic lung disease.
Lee	2023	Retrospective cohort study	Korea	NHIS^3^	NR^12^	7.52	ICD^4^-10	Treatment-based	342,601	ICD-10	86,233	NR	Children	Sex, calendar period of birth, birth season, region of residence, household income, breastfeeding, preterm birth, low birth weight, allergic rhinitis, asthma, diabetes mellitus, thyroid disorder, chronic inflammatory disease, chronic kidney disease, chronic neurological disorder, anemia, neuropsychiatric disorder, food allergy, and long-term use of systemic corticosteroids.
Matthewman	2022	Retrospective cohort study	UK	CPRD^5^ and HES^6^	1998–2016	4.41	Read code and ICD-10	Treatment-based	525,923	Read code and ICD-10	NR	2,562,334	NR	Age, sex, GP, date of cohort entry, calendar time, IMD, and asthma
Ha	2022	Retrospective cohort study	Korea	NHIS and NHSPIC^7^	NR	8.9	ICD-10	NR	38,319	ICD-10	NR	314,721	Children ≤71 months	Birth year, certain conditions originating during the perinatal period, physical examination results at 4–6 months old, clinical conditions with possible confounding effects.
Lin	2021	Retrospective cohort study	China	NHIRD^8^ and LHID^9^	1997–2013	NR	ICD-9	Healthcare utilization-based^14^	36,825	ICD-9	1,518	147,420	22.6 y	Each incremental year of age, sex, hospital visiting times, atopic dermatitis, moderate-to-severe atopic dermatitis, rheumatoid arthritis, osteoporosis, postmenopausal, topical corticosteroids, systemic corticosteroids, DMARDs, phototherapy.
Shaheen	2019	Retrospective cohort study	USA	NEDS^10^ and NIS^11^	2002–2012	NR	ICD-9	NR	NR	ICD-9	272,036	NR	≥ 50 y	NR
Lowe	2020	Retrospective cohort study	UK	CPRD and HES	1998–2016	5	NR	Treatment-based	526,808	NR	520,197	2,569,030	≥18 y	Time-updated asthma, IMD, and calendar time, BMI, smoking status, harmful alcohol use, and high-dose oral glucocorticoid use.
Wu	2017	Retrospective cohort study	China	NHIRD	/	NR	ICD-9	NR	35,229	BMD examination and ICD-9	360	35,229	20–49: 33.6 y	Age, sex, comorbidities (hypertension, diabetes mellitus, hyperlipidemia, chronic kidney disease, chronic liver disease, chronic obstructive pulmonary disease, depression) and use of systemic corticosteroids.
Garg	2015	Retrospective cohort study	USA	National Health and Nutrition Examination Survey	2005–2006	NR	Household surveys	NR	368	BMD examination and self-reported	33	4,604	20–85 y	NR

AD^1^: Atopic Dermatitis; BMD^2^: Bone Mineral Density; NHIS^3^: the National Health Insurance Service; ICD^4^: International Classification of Diseases; CPRD^5^: Clinical Practice Research Datalink; HES^6^: Hospital Episode Statistics; NHSPIC^7^: the National Screening Program for Infants and Children; NHIRD^8^: the National Health Insurance Research Database; LHID^9^: the Longitudinal Health Insurance Database; NEDS^10^: the Nationwide Emergency Department Sample; NIS^11^: the National Inpatient Sample; NR^12^: not reported; Treatment-based^13^: severity classified by medication type, phototherapy, or referral patterns; Healthcare utilization-based^14^: severity classified by hospitalization, medication duration, or visit frequency.

**Table 2.** The quality of included studies.

Study	Year	Selection	Comparability	Outcome	Total
Cohort studies (*n* = 10)					
Chiesa	2025	***	**	***	8
Garg	2015	**	*	**	5
Ha	2022	****	**	**	8
Hsiao	2024	***	**	***	8
Lee	2023	***	**	**	7
Lin	2021	***	**	**	7
Lowe	2020	****	*	**	7
Matthewman	2022	***	**	***	8
Shaheen	2019	***	**	***	8
Wu	2017	***	**	***	8

**Table 3.** Subgroup analysis for the risk of all-cause fractures in patients with AD.

Subgroups	Included studies	OR (95%CI)	Heterogeneity	
			*I* ^2^	*p* values
*AD severity*				
Mild AD	4	1.05 (1.02, 1.09)	95.2%	0.000
Moderate AD	3	1.08 (1.03, 1.12)	92.7%	0.000
Severe AD	3	1.26 (1.21, 1.31)	56.0%	0.059
Moderate to severe AD	2	1.23 (1.20, 1.26)	0.0%	0.526
*Sites*				
Cranial and facial bones	2	1.13 (1.10, 1.15)	0.0%	0.362
Lower limb bones	6	1.11 (1.08, 1.13)	65.0%	0.014
Ribs/thorax	1	0.97 (0.91, 1.03)		
Upper limb bones	5	1.06 (1.05, 1.07)	0.0%	0.571
Vertebral column	6	1.14 (1.08, 1.20)	67.3%	0.009

### Study selection

The initial set of records was imported into NoteExpress reference management software, and duplicate entries were removed to avoid redundancy. Two independent reviewers (Huilin Ru and Peng Yu) then performed title and abstract screening to exclude studies that were clearly irrelevant. The remaining records were classified as either eligible, ineligible, or uncertain. For studies with uncertain eligibility, full-text articles were reviewed to determine inclusion. Any disagreements between the two primary reviewers were resolved through discussion with a third reviewer (Shixuan Wang), ensuring consensus and minimizing bias in the selection process.

### Eligibility criteria

Studies were included in this meta-analysis if they investigated the association between AD and the risk of osteoporosis or fractures. Eligible studies needed to report outcomes such as diagnosed osteoporosis, all-cause fractures, or fractures at specific anatomical sites in participants diagnosed with AD or with a documented history of AD. The comparison group had to consist of healthy individuals or non-AD patients. Furthermore, the studies were required to provide effect estimates such as odds ratios (OR), relative risks (RR), or 95% confidence intervals (CIs), which allowed for the assessment of the strength of the association between AD and the specified outcomes. Only cohort studies, either prospective or retrospective, were included to minimize methodological biases that could arise from other types of observational studies. When multiple publications used the same underlying database over overlapping time periods, we prioritized the cohort with the largest sample size and most comprehensive reporting for the primary meta-analysis and used additional overlapping studies only for sensitivity or subgroup analyses to minimize double-counting of participants.

Studies were excluded if they did not meet the above criteria, such as conference abstracts, commentaries, or duplicate publications. Also, studies that did not report independent data on fractures or osteoporosis, like reviews or meta-analyses without primary data, were excluded. This ensured that only high-quality, relevant studies were included, providing the most reliable evidence on the relationship between AD and fracture or osteoporosis risk.

### Data extraction

Data were systematically extracted using a pre-designed form created in Microsoft Excel (Microsoft Corporation, USA). Two reviewers (Hongfei Liu and Wei Wei) independently extracted data from eligible studies, including the following key information: first author, year of publication, study type, country of origin, data source, enrollment period, follow-up duration, diagnostic criteria for AD, the number of AD patients, fracture/osteoporosis diagnostic criteria, number of fracture cases, size of the control group, patient age, and potential confounders. Extracted data were cross-verified to ensure accuracy and consistency, and any discrepancies were resolved through discussion and consensus.

### Study quality

The methodological quality of the included cohort studies was evaluated using the Newcastle-Ottawa Quality Assessment Scale (NOS) (available at: http://www.ohri.ca/programs/clinical_epidemiology/oxford.asp). The NOS assesses three major domains: selection, comparability, and outcome assessment, with a total score ranging from 0 to 9 stars. Studies were categorized based on their NOS score as follows: high quality (7–9 stars), moderate quality (4–6 stars), and low quality (0–3 stars).

### Data synthesis

Data analysis was performed using Stata software (version 14). Due to anticipated clinical, methodological, and statistical heterogeneity across studies, a random-effects model was employed to pool the effect estimates, enhancing the robustness and generalizability of the findings [19,20]. Sensitivity analyses were conducted to evaluate the stability of the overall results, helping to identify potential sources of heterogeneity. Subgroup analyses were performed to explore variations in fracture risk based on factors such as fracture site and the severity of AD. Publication bias was evaluated using funnel plots and Egger’s regression test.

## Results

### Study selection

**Figure 1.**
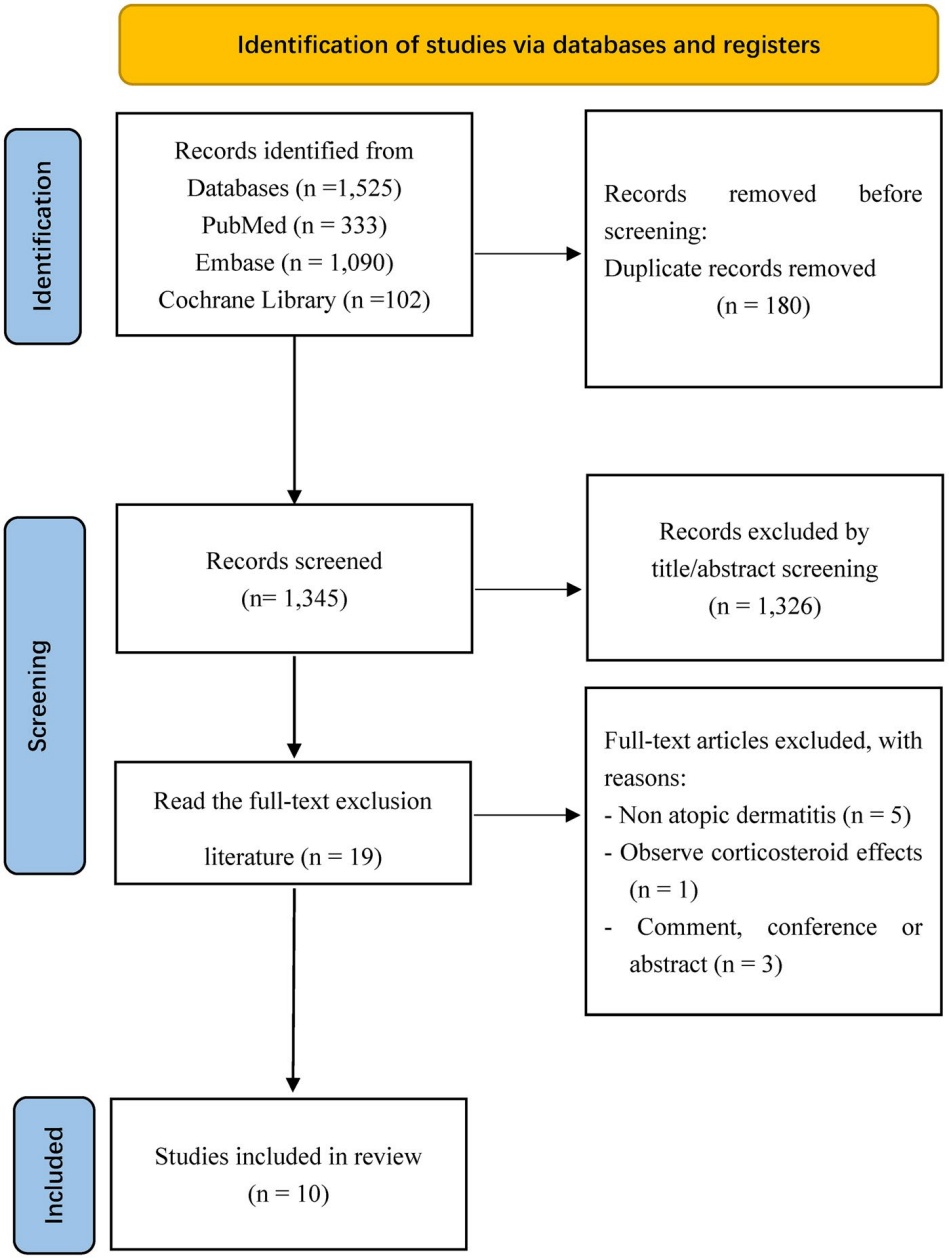
Literature screening flow chart.

A total of 1,525 records were identified from various databases, including PubMed (*n* = 333), Embase (*n* = 1,090), and the Cochrane Library (*n* = 102). After removing duplicates (*n* = 180), 1,345 records remained for screening. From the title and abstract screening, 1,326 records were excluded, leaving 19 full-text articles for further evaluation. These full-text articles were excluded for the following reasons: non-atopic dermatitis (*n* = 5), observational corticosteroid effects (*n* = 1), and being comments, conference abstracts, or similar (*n* = 3). The list of excluded studies is in Supplementary Table 4. Ultimately, 10 studies [13–16, 21–25, 27] were included in the meta-analysis. This process is visually depicted in the literature screening flowchart (Figure 1).

### Studies characteristics

This meta-analysis includes 10 cohort studies conducted in various countries, including the UK, China, Korea, the USA, and Taiwan, from 2005 to 2025. These studies analyzed the relationship between AD and the risk of fractures or osteoporosis, utilizing diverse data sources such as national health databases, hospital records, and household surveys. The diagnostic criteria for AD and fractures/osteoporosis varied across studies, with some using ICD codes, medical records, and bone mineral density (BMD) examinations. The patient populations included both children and adults, with sample sizes ranging from 368 to over 2 million participants. Follow-up periods ranged from 4 to 9 years, with adjustment for multiple confounders such as age, BMI, smoking, comorbidities, and medication use. The basic characteristics of the included cohorts are shown in Table 1.

### Quality of included studies

Most studies received a total score between 7 and 8, indicating generally good quality. The selection domain was strong for most studies, with several studies achieving high ratings. However, the comparability domain showed some variability, with a few studies scoring lower, reflecting weaker control of confounders. In the outcome domain, most studies demonstrated adequate methods for assessing outcomes. Overall, the studies were of good methodological quality, with minor weaknesses in controlling for confounders in some studies. The detailed assessment scores are presented in Table 2.

### AD and the risk of all-cause fractures

**Figure 2.**
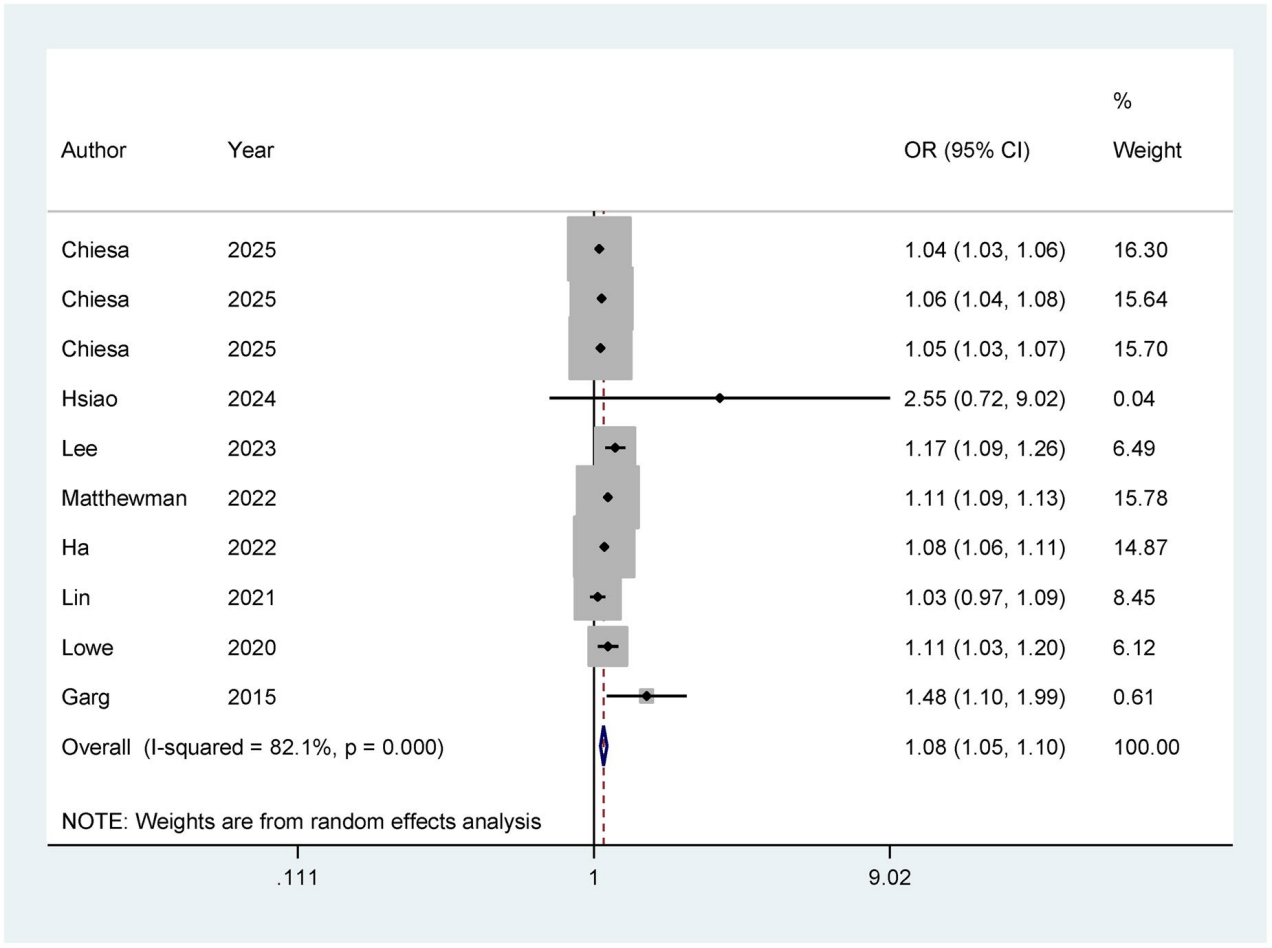
Meta-analysis of AD and the risk of all-cause fractures.

The overall pooled analysis shows a significant association, with an OR of 1.08 (95% CI: 1.05, 1.10), suggesting that patients with AD have an increased risk of fractures compared to those without AD. The heterogeneity is high (*I*^2^ = 82.1%), indicating substantial variability between the studies (Figure 2). The sensitivity analysis plot provides a visual representation of the influence of individual studies on the overall effect estimate. It shows that the meta-analysis results remain robust even when specific studies are omitted. The individual study estimates, represented by their confidence intervals, vary but consistently suggest an increased fracture risk in AD patients. This indicates the robustness of the findings across different study populations and methodologies (Supplementary Figure 1).

### AD and the risk of osteoporosis

**Figure 3.**
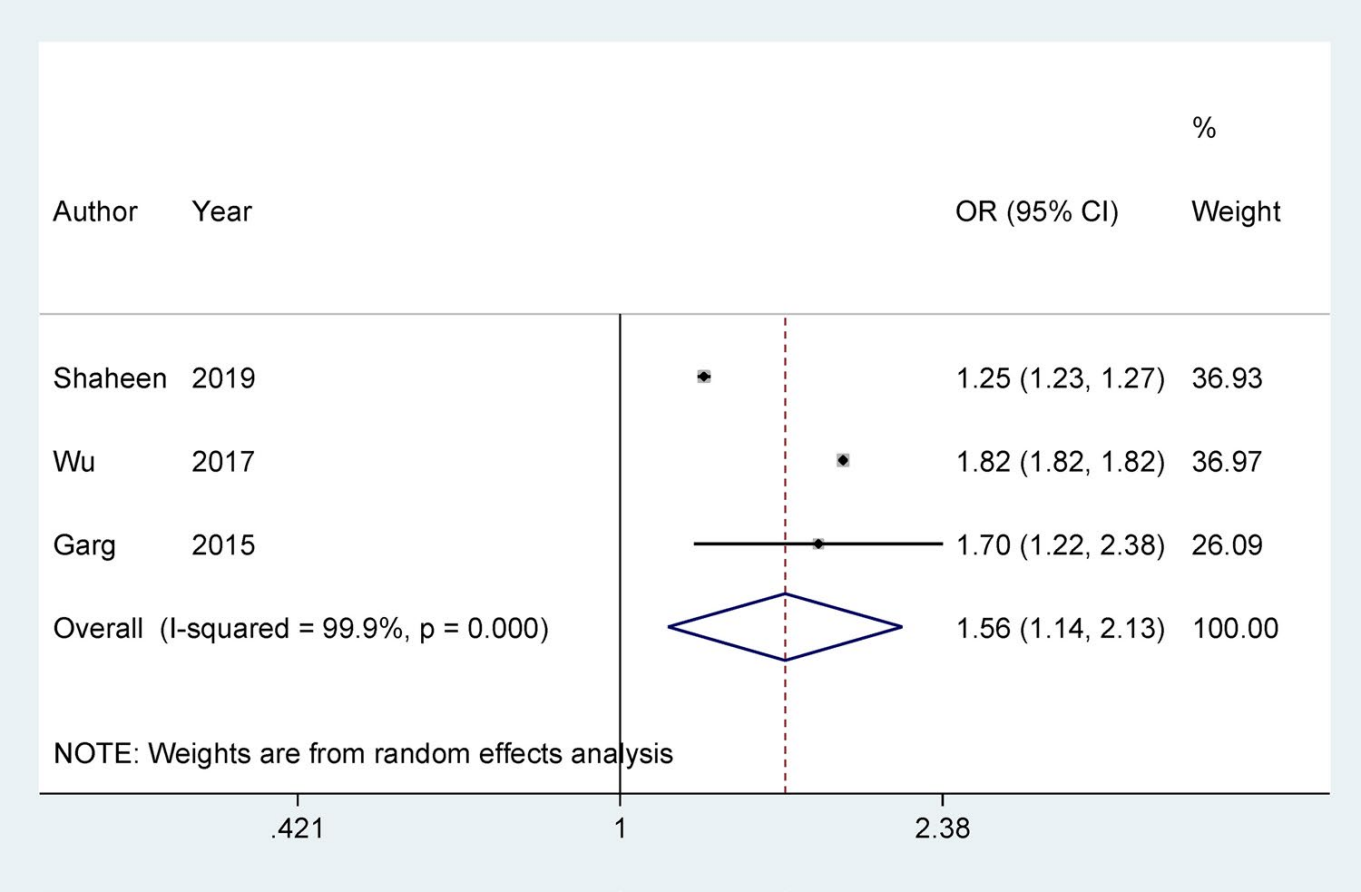
Meta-analysis of AD and the risk of osteoporosis.

The pooled OR for osteoporosis risk in AD patients is 1.56 (95% CI: 1.14, 2.13), suggesting a significant increase in osteoporosis risk among AD patients. The heterogeneity of the studies is very high, with an I^2^ value of 99.9% (*p* = 0.000), indicating substantial variability between the included cohort studies (Figure 3). The sensitivity analysis, shown in Supplementary Figure 2, assesses the robustness of the overall findings by excluding one study at a time. The results remain consistent even when individual studies are omitted, indicating the robustness of the meta-analysis. This suggests that the relationship between AD and the increased risk of osteoporosis is stable across different studies.

### Subgroup analysis

For disease severity, the analysis reveals that the risk of fractures increases with the severity of AD. Mild AD is associated with a OR of 1.05 (95% CI: 1.02, 1.09), with high heterogeneity (*I*^2^ = 95.2%, *p* < 0.0001). Moderate AD shows a slightly higher OR of 1.08 (95% CI: 1.03, 1.12) with similarly high heterogeneity (*I*^2^ = 92.7%, *p* < 0.0001). Severe AD has an even higher OR of 1.26 (95% CI: 1.21, 1.31), but with moderate heterogeneity (*I*^2^ = 56.0%, *p* = 0.059). For moderate to severe AD, the OR is 1.23 (95% CI: 1.20, 1.26), showing no significant heterogeneity (*I*^2^ = 0.0%, *p* = 0.526), suggesting consistent results across studies.

In terms of fracture sites, the risk is highest for vertebral column, with an OR of 1.14 (95% CI: 1.08, 1.20) and moderate heterogeneity (*I*^2^ = 67.3%, *p* = 0.009). For lower limb bones, the OR is 1.11 (95% CI: 1.08, 1.13) with moderate heterogeneity (*I*^2^ = 65.0%, *p* = 0.014). The rib/thorax site shows no significant association (OR = 0.97, 95% CI: 0.91, 1.03), with no data on heterogeneity. The upper limb bones show a slight increase in risk (OR = 1.06, 95% CI: 1.05, 1.07) with no heterogeneity (*I*^2^ = 0.0%, *p* = 0.571). Lastly, fractures of the cranial and facial bones are associated with an OR of 1.13 (95% CI: 1.10, 1.15), with no heterogeneity (*I*^2^ = 0.0%, *p* = 0.362). The subgroup analysis for the risk of all-cause fractures in patients with AD is summarized in Table 3.

### Publication bias

**Figure 4.**
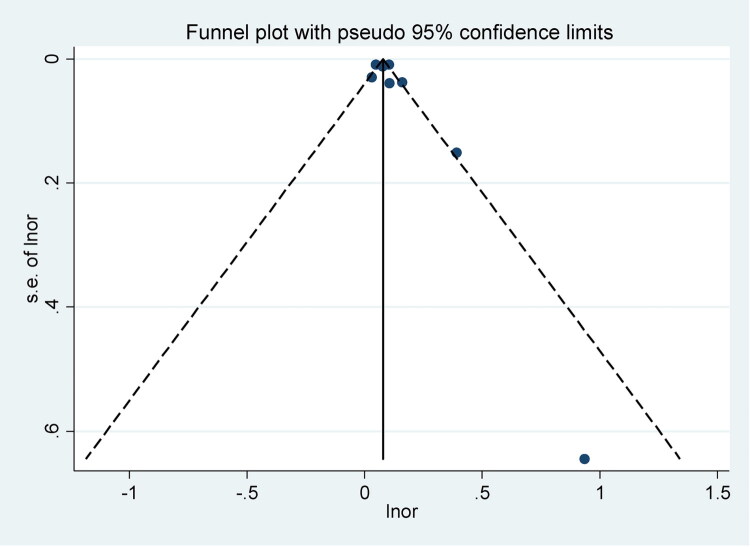
Funnel plot of publication bias.

The funnel plot appears symmetrical, suggesting no significant publication bias in the included studies (Figure 4). Additionally, Egger’s test was conducted to statistically assess publication bias, with a *p* value of 0.316. This *p* value indicates that there is no evidence of publication bias, as the result is not statistically significant (*p* > 0.05).

## Discussion

### Main findings

This meta-analysis provides a comprehensive review of the risk of osteoporosis and fractures in patients with atopic dermatitis (AD), comparing them to those without AD, based on data from ten cohort studies. We found a significant association between AD and increased risks of osteoporosis and fractures. Specifically, AD patients showed a 56% higher risk of osteoporosis (OR = 1.56, 95% CI: 1.14–2.13; *I*^2^ = 99.9%, *p* < 0.0001) and an 8% higher risk of all-cause fractures (OR = 1.08, 95% CI: 1.05–1.10; *I*^2^ = 82.1%, *p* < 0.0001). Subgroup analyses further revealed that fracture risk increased progressively with the severity of AD. Site-specific fracture risks were significantly higher for the vertebral column (OR = 1.14, 95% CI: 1.08–1.20; *I*^2^ = 67.3%, *p* = 0.009) and the lower limb (OR = 1.11, 95% CI: 1.08–1.13; *I*^2^ = 65.0%, *p* = 0.014). These findings reinforce previous research on the relationship between AD and skeletal complications while contributing new insights, especially by quantifying fracture risks in different body regions.

### Comparison with previous meta-analyses

Our results are consistent with previous studies [11,12], confirming significantly increased risks of both osteoporosis (OR = 1.56 vs. RR = 1.95 in Wu et al.) and all-cause fractures (OR = 1.08 vs. RR = 1.13 in Wu et al.), solidifying AD as an independent risk factor for these outcomes. However, our study improves on prior work by incorporating methodological advancements. Previous meta-analyses included both cross-sectional (*n* = 7) and cohort studies (*n* = 3), which may have introduced susceptibility bias. Moreover, Egger’s regression test indicated publication bias (*p* = 0.028). In contrast, we focused exclusively on cohort studies (*n* = 10), reducing heterogeneity and enhancing causal inference, with no evidence of publication bias (*p* = 0.316). Furthermore, our analysis uniquely quantified risks for vertebral and lower limb fractures (OR = 1.14, *p* = 0.009 and OR = 1.11, *p* = 0.014, respectively), a novel contribution. Additionally, we identified a progressive increase in fracture risk with greater AD severity, emphasizing the need for tailored clinical approaches for patients with severe AD, especially those at risk for vertebral and lower limb fractures.

### Possible mechanisms linking atopic dermatitis and bone health

The association between AD and bone health can be explained by multiple factors related to the pathophysiology of AD and its treatments. AD is a chronic inflammatory condition [2], and the activation of osteoclasts by pro-inflammatory cytokines such as IL-1, IL-6, IL-17, IL-31, IL-33, TNF-α, and RANKL accelerates bone resorption [26]. This inflammatory process may contribute to the early onset of bone loss, particularly in children and adolescents [15,27]. Additionally, glucocorticoids (GCs), commonly prescribed for AD, can impair bone formation and enhance resorption through various mechanisms, including alterations in muscle strength, calcium and vitamin D metabolism, fat metabolism, and sex steroid levels [28,29]. While systemic and topical glucocorticoids are well-established contributors to bone loss, one large cohort reported that the association between AD and fractures persisted after adjustment for oral corticosteroid use, suggesting that corticosteroids alone do not fully explain the increased risk [16]. Lifestyle and dietary factors may also play a role, such as reduced calcium and vitamin D intake due to food intolerances, lower physical activity due to itching, and psychological factors like stress and sleep disturbances [7,11,16,27]. Moreover, our previous Mendelian randomization study suggested a potential causal relationship between allergic diseases, eosinophils, and osteoporosis based on genetic variation [8]. In summary, the link between AD and bone health is complex, extending beyond the effects of GCs and inflammation, and warrants further investigation.

## Limitations and clinical implications

This meta-analysis adheres to the latest PRISMA 2020 guidelines, was prospectively registered with PROSPERO, and includes the largest cohort of patients with AD examined in relation to osteoporosis and fractures. These findings offer valuable insights for clinical practice, particularly for the prevention and management of bone health in AD patients.

However, several limitations must be acknowledged. First, substantial heterogeneity was observed in the pooled estimates, especially for osteoporosis (*I*^2^ = 99.9%) and all-cause fractures (*I*^2^ = 82.1%). This variability may be due to differences in study populations (e.g. age, sex), data sources (e.g. administrative vs. clinical databases), diagnostic criteria for AD and outcomes (e.g. ICD codes vs. BMD measurements), and the degree of confounder adjustment. Although sensitivity and subgroup analyses confirmed the robustness of our results, unexplained heterogeneity limits the precision and generalizability of our findings. Second, while our stratified analysis by AD severity showed a gradient in fracture risk, many studies lacked standardized definitions of AD severity, potentially introducing misclassification bias. Future studies should use standardized scoring systems (e.g. SCORAD, EASI) to enhance comparability. Third, information on topical and systemic corticosteroid exposure was not consistently reported across the included cohorts. Although several studies adjusted for or stratified by oral and/or topical corticosteroid use, others did not provide sufficient detail to allow pooled analyses by corticosteroid dose or duration. Therefore, residual confounding by corticosteroid exposure cannot be entirely excluded. Moreover, important determinants of bone health, including physical activity, diet, sun exposure, vitamin D or calcium supplements, smoking, and alcohol use, were not uniformly measured. These unmeasured or incompletely captured factors may influence both AD severity and bone outcomes, and thus our results should be interpreted as evidence of association rather than proof of causality. Fourth, diagnostic bias is an inherent limitation of observational studies using routine healthcare data. Patients with AD are more likely to have repeated contact with healthcare providers, which may increase the probability of diagnosing both AD and comorbid osteoporosis or fractures. Because the included studies relied on routine medical records, differential healthcare utilization may introduce potential diagnostic or surveillance bias that cannot be fully eliminated. Lastly, as all studies included were retrospective cohort studies, causal inference is limited by potential bias in exposure and outcome assessment. Prospective studies are needed to validate these findings.

In addition to adults, children with AD also have a relatively high incidence of fracture events. From a clinical perspective, fractures in children may affect peak bone mass and future bone development, while osteoporosis and fractures in adults (especially the elderly) are major factors contributing to morbidity, disability, and mortality. Therefore, identifying age-specific risks associated with traumatic fractures is crucial for formulating precise prevention strategies tailored to children and adult populations. Because few paediatric cohorts reported age-stratified effect estimates, future large studies should provide separate risk estimates for children, adolescents, and adults to allow more refined age-specific meta-analyses.

Furthermore, the included cohort studies did not consistently report baseline fracture or osteoporosis incidence in AD versus non-AD groups, preventing a reliable pooled calculation of absolute risk differences. Despite this limitation, even small relative increases may lead to meaningful public health impacts given the high global prevalence of AD. Importantly, our findings still warrant clinical attention, as they highlight the need for greater vigilance for bone health in AD populations. Specifically, our results suggest that AD (particularly moderate-to-severe disease, older age, and concomitant long-term systemic corticosteroid use) may be considered a risk-enhancing factor when evaluating the need for osteoporosis screening and fracture prevention. In such patients, clinicians may reasonably prioritize bone-health counseling (adequate calcium and vitamin D intake, safe weight-bearing exercise), cautious use of systemic corticosteroids, and consideration of bone mineral density testing. From the perspective of healthcare services, this may be more feasible than universal screening. However, considering Alzheimer’s disease as a risk marker in existing fracture risk assessment approaches requires further evaluation of its cost-effectiveness in dedicated health economics research.

## Conclusion

This meta-analysis provides evidence that AD is associated with an increased risk of osteoporosis and fractures, particularly in patients with severe AD and those at higher risk of vertebral or lower limb fractures. These findings highlight the importance of targeted bone health monitoring in the clinical management of AD. However, due to the heterogeneity and potential residual confounding, clinicians should interpret these findings with caution.

Future prospective studies are warranted to more rigorously examine the association between AD and adverse bone outcomes. Such studies should incorporate standardized assessments of AD severity and bone health, using validated severity scoring systems and uniform diagnostic criteria for osteoporosis and fractures. They should also include broader representation of pediatric patients, enable long-term follow-up from childhood-onset AD into adulthood, and systematically document corticosteroid exposure and other key determinants of bone health. These efforts will help clarify the underlying associations and support the development of more precise and individualized prevention strategies for patients with AD.

## Supplementary Material

Supplemental Material

Appendix PRISMA_2020_checklist.docx

## Data Availability

Data are available from the corresponding author upon reasonable request.

## References

[1] Ständer S. Atopic dermatitis. N Engl J Med. 2021;384(12):1136–1143. doi: 10.1056/NEJMra2023911. 33761208

[2] Guttman-Yassky E, Renert-Yuval Y, Brunner PM. Atopic dermatitis. Lancet. 2025;405(10478):583–596. doi: 10.1016/S0140-6736(24)02519-4. 39955121

[3] GBD 2021 Asthma and Allergic Diseases Collaborators. Global, regional, and national burden of asthma and atopic dermatitis, 1990-2021, and projections to 2050: a systematic analysis of the Global Burden of Disease Study 2021. Lancet Respir Med. 2025;13(5):425–446. doi: 10.1016/S2213-2600(25)00003-7. 40147466

[4] Schuler CF 4th, Billi AC, Maverakis E, et al. Novel insights into atopic dermatitis. J Allergy Clin Immunol. 2023;151(5):1145–1154. doi: 10.1016/j.jaci.2022.10.023. 36428114 PMC10164702

[5] Weidinger S, Beck LA, Bieber T, et al. Atopic dermatitis. Nat Rev Dis Primers. 2018;4(1):1; [cited 2018 Jun 21]. doi: 10.1038/s41572-018-0001-z. 29930242

[6] Thyssen JP, Halling AS, Schmid-Grendelmeier P, et al. Comorbidities of atopic dermatitis – what does the evidence say? J Allergy Clin Immunol. 2023;151(5):1155–1162. doi: 10.1016/j.jaci.2022.12.002. 36621338

[7] Mukovozov IM, Morra DE, Giustini D, et al. Atopic dermatitis and bone health: a systematic review. J Eur Acad Dermatol Venereol. 2021;35(3):615–628. doi: 10.1111/jdv.16895. 32853421

[8] Yue X, Liu H, Yang S, et al. Causal association of allergic diseases, eosinophils, and osteoporosis: a Mendelian randomization study. World Allergy Organ J. 2025;18(3):101039; [cited 2025 Mar 11]. doi: 10.1016/j.waojou.2025.101039. 40151544 PMC11946878

[9] Jang YH, Choi EY, Lee H, et al. Long-term use of oral corticosteroids and safety outcomes for patients with atopic dermatitis. JAMA Netw Open. 2024;7(7):e2423563. doi: 10.1001/jamanetworkopen.2024.23563. 39028668 PMC11259904

[10] Yang TH, Chen PC, Lin YC, et al. Adolescents with atopic dermatitis have lower peak exercise load capacity and exercise volume compared with unaffected peers. Int J Environ Res Public Health. 2022;19(16):10285; [cited 2022 Aug 18]. doi: 10.3390/ijerph191610285. 36011919 PMC9407882

[11] Wu D, Wu XD, Zhou X, et al. Bone mineral density, osteopenia, osteoporosis, and fracture risk in patients with atopic dermatitis: a systematic review and meta-analysis. Ann Transl Med. 2021;9(1):40–40. doi: 10.21037/atm-20-4708. 33553333 PMC7859773

[12] Zhou B, Liang S, Shang S, et al. Evaluation of bone fracture risks in patients with atopic dermatitis: meta-analysis and trial sequential analysis. Postepy Dermatol Alergol. 2023;40(5):699–701. doi: 10.5114/ada.2023.132244. 38028422 PMC10646716

[13] Chiesa Fuxench ZC, Wan J, Wang S, et al. Fracture risk among adults with atopic dermatitis: a population-based cohort study in the United Kingdom. J Eur Acad Dermatol Venereol. 2025;039(9):e816–e819. doi: 10.1111/jdv.20728. 40353612

[14] Hsiao YY, Chen YH, Chen YW, et al. The fracture risk of elderly patients with atopic dermatitis. Dermatitis. 2024;35(4):380–385. doi: 10.1089/derm.2023.0174. 38227792

[15] Lee SW, Shin YH, Shin JI, et al. Fracture incidence in children after developing atopic dermatitis: a Korean nationwide birth cohort study. Allergy. 2023;78(3):871–875. doi: 10.1111/all.15577. 36353744

[16] Matthewman J, Mansfield KE, Prieto-Alhambra D, et al. Atopic eczema-associated fracture risk and oral corticosteroids: a population-based cohort study. J Allergy Clin Immunol Pract. 2022;10(1):257–266.e8. doi: 10.1016/j.jaip.2021.09.026. 34571200 PMC7612204

[17] Page MJ, McKenzie JE, Bossuyt PM, et al. The PRISMA 2020 statement: an updated guideline for reporting systematic reviews. BMJ. 2021;372:n71; [cited 2021 Mar 29]. doi: 10.1136/bmj.n71. 33782057 PMC8005924

[18] Shea BJ, Reeves BC, Wells G, et al. AMSTAR 2: a critical appraisal tool for systematic reviews that include randomised or non-randomised studies of healthcare interventions, or both. BMJ. 2017;358:j4008. doi: 10.1136/bmj.j4008. 28935701 PMC5833365

[19] Xie W, Wang Y, Xiao S, et al. Association of gestational diabetes mellitus with overall and type specific cardiovascular and cerebrovascular diseases: systematic review and meta-analysis. BMJ. 2022;378:e070244. doi: 10.1136/bmj-2022-070244. 36130740 PMC9490552

[20] Fu K, Wang J, Pan H, et al. Weekend warrior and the risk of specific disease: a meta-epidemiology study. BMC Public Health. 2025;25(1):1414. doi: 10.1186/s12889-025-22667-7. 40234866 PMC11998413

[21] Lin TL, Wu CY, Yen JJ, et al. Fracture risks in patients with atopic dermatitis: a nationwide matched cohort study. Ann Allergy Asthma Immunol. 2021;127(6):667–673.e2. doi: 10.1016/j.anai.2021.09.004. 34537357

[22] Shaheen MS, Silverberg JI. Atopic dermatitis is associated with osteoporosis and osteopenia in older adults. J Am Acad Dermatol. 2019;80(2):550–551. doi: 10.1016/j.jaad.2018.05.026. 29800580

[23] Lowe KE, Mansfield KE, Delmestri A, et al. Atopic eczema and fracture risk in adults: a population-based cohort study. J Allergy Clin Immunol. 2020;145(2):563–571.e8. doi: 10.1016/j.jaci.2019.09.015. 31757515 PMC7014587

[24] Wu CY, Lu YY, Lu CC, et al. Osteoporosis in adult patients with atopic dermatitis: a nationwide population-based study. PLoS One. 2017;12(2):e0171667. doi: 10.1371/journal.pone.0171667. 28207767 PMC5313211

[25] Garg NK, Silverberg JI. Eczema is associated with osteoporosis and fractures in adults: a US population-based study. J Allergy Clin Immunol. 2015;135(4):1085–1087.e2. doi: 10.1016/j.jaci.2014.10.043. 25512080

[26] Sirufo MM, De Pietro F, Bassino EM, et al. Osteoporosis in skin diseases. Int J Mol Sci. 2020;21(13):4749; [cited 2020 Jul 03]. doi: 10.3390/ijms21134749. 32635380 PMC7370296

[27] Ha EK, Kim JH, Kwak JH, et al. Association of clinical and social factors with risk of fracture in children with atopic dermatitis. Pediatr Allergy Immunol. 2022;33(2):e13712. doi: 10.1111/pai.13712. 34862671

[28] Hardy RS, Zhou H, Seibel MJ, et al. Glucocorticoids and bone: consequences of endogenous and exogenous excess and replacement therapy. Endocr Rev. 2018;39(5):519–548. doi: 10.1210/er.2018-00097. 29905835

[29] Chen M, Fu W, Xu H, et al. Pathogenic mechanisms of glucocorticoid-induced osteoporosis. Cytokine Growth Factor Rev. 2023;70:54–66. doi: 10.1016/j.cytogfr.2023.03.002. 36906448 PMC10518688

