# Supplementary material for: Atopic dermatitis and the risk of osteoporosis and fractures: a meta-analysis of cohort studies
Source: Ann Med. 2026 Jan 8;58(1):2607193. doi: 10.1080/07853890.2025.2607193 (PMC12794696; doi:10.1080/07853890.2025.2607193)
Supplement: Supplemental Material [file IANN_A_2607193_SM8245.docx]

Supplementary Table 1 PubMed 2025.05.30

| No. | Query | Results |
| --- | --- | --- |
| 1 | "Dermatitis, Atopic"[Mesh] | 27,307 |
| 2 | (Dermatitis*[Title/Abstract]) OR (Neurodermatitis*[Title/Abstract]) | 81,106 |
| 3 | ("Dermatitis, Atopic"[Mesh]) OR ((Dermatitis*[Title/Abstract]) OR (Neurodermatitis*[Title/Abstract])) | 86,985 |
| 4 | ("Osteoporosis"[Mesh]) OR "Fractures, Bone"[Mesh] | 269,628 |
| 5 | (((Osteoporos*[Title/Abstract]) OR (Bone Loss*[Title/Abstract])) OR (Fracture*[Title/Abstract])) OR (Broken Bone*[Title/Abstract]) | 429,648 |
| 6 | (("Osteoporosis"[Mesh]) OR "Fractures, Bone"[Mesh]) OR ((((Osteoporos*[Title/Abstract]) OR (Bone Loss*[Title/Abstract])) OR (Fracture*[Title/Abstract])) OR (Broken Bone*[Title/Abstract])) | 480,314 |
| 7 | (("Dermatitis, Atopic"[Mesh]) OR ((Dermatitis*[Title/Abstract]) OR (Neurodermatitis*[Title/Abstract]))) AND ((("Osteoporosis"[Mesh]) OR "Fractures, Bone"[Mesh]) OR ((((Osteoporos*[Title/Abstract]) OR (Bone Loss*[Title/Abstract])) OR (Fracture*[Title/Abstract])) OR (Broken Bone*[Title/Abstract]))) | 333 |

Supplementary Table 2 Embase 2025.05.30

| No. | Query | Results |
| --- | --- | --- |
| #1 | 'atopic dermatitis'/exp | 67519 |
| #2 | dermatitis*:ab,ti OR neurodermatitis*:ab,ti | 113473 |
| #3 | #1 OR #2 | 133575 |
| #4 | 'osteoporosis'/exp | 173450 |
| #5 | 'fracture'/exp | 431995 |
| #6 | 'broken bone*':ab,ti OR 'bone loss*':ab,ti OR osteoporos*:ab,ti OR fracture*:ab,ti | 535336 |
| #7 | #4 OR #5 OR #6 | 688143 |
| #8 | #3 AND #7 | 1090 |

Supplementary Table 3 Cochrane Library 2025.05.30

| No. | Query | Results |
| --- | --- | --- |
| #1 | MeSH descriptor: [Dermatitis, Atopic] explode all trees | 2747 |
| #2 | (Dermatitis*):ti,ab,kw OR (Neurodermatitis*):ti,ab,kw (Word variations have been searched) | 12110 |
| #3 | #1 OR #2 | 12110 |
| #4 | MeSH descriptor: [Osteoporosis] explode all trees | 5513 |
| #5 | MeSH descriptor: [Fractures, Bone] explode all trees | 9630 |
| #6 | (Osteoporos*):ti,ab,kw OR (Bone Loss*):ti,ab,kw OR (Fracture*):ti,ab,kw OR (Broken Bone*):ti,ab,kw (Word variations have been searched) | 47144 |
| #7 | #4 OR #5 OR #6 | 47179 |
| #8 | #3 AND #7 | 102 |

Supplementary Table 4. Literature exclusion list

| No. | Title | Journal |
| --- | --- | --- |
| *Non atopic dermatitis (n=5)* | | |
| 1 | No increase in risk of fracture, malignancy or mortality in dermatitis herpetiformis: a cohort study | Aliment Pharmacol Ther |
| 2 | Dermatitis herpetiformis and bone mineral density: Analysis of a French cohort of 53 patients | European Journal of Dermatology |
| 3 | Impact of seborrheic dermatitis on osteoporosis risk: A population-based cohort study | The Journal of dermatology |
| 4 | Risk of fractures in dermatitis herpetiformis and coeliac disease: a register-based study | Scand J Gastroenterol |
| 5 | Self-Reported Fractures in Dermatitis Herpetiformis Compared to Coeliac Disease | Nutrients |
| *Observe corticosteroid effects（n=1）* | | |
| 1 | Association between topical corticosteroid use and fracture risk among pediatric patients with atopic dermatitis | Journal of the American Academy of Dermatology |
| *Comment, conference, or abstract (n=3)* | | |
| 1 | Vertebral, pelvic, and hip fracture risk in adults with severe atopic dermatitis | J Allergy Clin Immunol |
| 2 | Atopic eczema associated fracture risk and oral corticosteroids: A population-based cohort study | Pharmacoepidemiology and Drug Safety |
| 3 | 185 Fracture risk in adult and pediatric patients with atopic dermatitis -a population-based cohort study | Journal of Investigative Dermatology |

Supplementary Figure1 Sensitivity analysis of AD and the forest map of all-cause fracture risk

Supplementary Figure 2: Sensitivity analysis of AD and the meta-analysis of osteoporosis risk
